# Participation of the histamine receptor encoded by the gene *hclB* (HCLB) in visual sensitivity control: an electroretinographic study in *Drosophila melanogaster*

**Published:** 2012-10-06

**Authors:** Petia Kupenova, Shazie Yusein-Myashkova

**Affiliations:** 1 Department of Physiology, Medical University, Sofia, Bulgaria; 2 Institute of Molecular Biology, Bulgarian Academy of Sciences, Sofia, Bulgaria

## Abstract

**Purpose:**

Histaminergic transmission in the first synapse of the visual system in *Drosophila melanogaster* is mediated by two types of histamine receptors: 1) encoded by the gene *hclA* (HCLA), which is expressed in the second-order neurons—the large monopolar cells of the lamina, and is absolutely required for forward signal transmission; and 2) encoded by the gene *hclB* (HCLB), which is expressed in epithelial glia, and is involved in modulation of synaptic transmission from photoreceptors to large monopolar cells. The aim of our study was to establish whether the HCLB receptor–mediated modulation of synaptic transmission 1) contributes to the process of light adaptation, and 2) is involved in the control of the dynamics of sensitivity recovery after short-term light adaptation.

**Methods:**

The effects of mutations in the gene *hclB*, encoding the subunits of the histamine receptor HCLB, were studied on 1) the intensity-response (V/logI) function of electroretinographic (ERG) responses under dark adaptation, as well as under three levels of background illumination; and 2) the dynamics of the dark sensitivity recovery after short-term light adaptation.

**Results:**

The amplitude of the photoreceptor component in the electroretinogram (ERG) was not significantly different between the *hclB* mutants and the wild-type flies, while the amplitude of the ERG ON and OFF transients, representing the activity of the second-order visual cells, was increased in the *hclB* mutants under both dark and light adaptation. The ON responses were affected to a greater degree. Under a given background, the ON response V/logI function was steeper and the response dynamic range was narrowed. The absolute sensitivity of the two transients was increased, as revealed by the decrease of their thresholds. The relative sensitivity of the transients, assessed by the semisaturation points of their V/logI functions, was decreased in ON responses to long (2 s) stimuli under dark and moderate light adaptation, being unchanged under bright backgrounds. Thus, the shift of the ON response V/logI function along the stimulus intensity axis during light adaptation occurred within a narrower range. The peak latencies of the ERG transients were delayed. The slower kinetics of the ERG transients was also indicated by their lower sensitivity to low-pass filtering, the effect being more pronounced under light adaptation. In wild-type flies, an instant dark sensitivity recovery or postadaptational potentiation of the ERG transients was usually observed after short-term light adaptation. In the *hclB* mutants the dark sensitivity recovery in similar conditions was significantly delayed.

**Conclusions:**

The glial histamine receptor HCLB participates in visual sensitivity control at the level of the first synapse of the *Drosophila* visual system under a wide range of ambient illumination conditions and contributes to the process of light adaptation. The HCLB receptor-mediated modulation of synaptic gain helps avoid response saturation and increases the range of stimulus intensities within which dynamic responses can be generated. The HCLB receptors also speed up the sensitivity recovery after short-term light adaptation and contribute to the mechanism of postadaptational potentiation. They modulate the temporal characteristics of visual responses in a way that improves the temporal resolution of the visual system and reduces redundant (low-frequency) information.

## Introduction

Histamine is the neurotransmitter of the photoreceptors in the visual system of arthropods [1-5]. It exerts a hyperpolarizing action on the postsynaptic neurons, mediated through ligand-gated Cl¯ channels [6,7]. In *Drosophila*, two genes—histamine-gated chloride channel subunit A (*hclA*) and histamine-gated chloride channel subunit B (*hclB*; according to the nomenclature in [8])—encode histamine receptor subunits [8-11]. The expression of the two genes was studied using a reporter gene strategy [12,13] and mRNA tagging technique [12]. In the first optic neuropile of the *Drosophila* visual system, the lamina, expression of HCLA was proved in the neurons postsynaptic to the R1-R6-type photoreceptors, namely, the large monopolar cells (LMCs), and possibly the amacrine cells [12,13]. In contrast, expression of HCLB was only found in the epithelial glia [12,13]. The epithelial glia separates the individual cartridges in the lamina, within which R1-R6-type photoreceptors contact second-order cells, participating in the creation of a high resistance barrier [14-16]. Along with LMCs, the epithelial glia is a postsynaptic element in the tetrad synapses, formed by R1-R6-type photoreceptors [14,16,17].

Mutations in *hclA* (the mutants also known as ora transientless [*ort*]) abolish signal transmission from photoreceptors to LMCs, which is indicated by the changes in the electroretinogram (ERG) [8]. In the ERG of the *ort* mutants, the photoreceptor (sustained) component is preserved, while the postreceptoral ones, the ON and OFF transients originating from the LMCs [18-20], are lacking [8]. In contrast, in the *hclB* mutants, the amplitude of the ERG ON [12] or ON and OFF [21,22] transients is increased and the time course of their LMCs responses is delayed [12]. The increased amplitude of the ERG transients in the *hclB* mutants, with no significant change in the photoreceptor component, indicates that in these mutants, the gain of synaptic transmission from photoreceptors to LMCs is increased. In spite of their glial location, the HCLB receptors are capable of affecting synaptic gain and shaping the amplitude and time course of LMC responses by changing the parameters of the synaptic environment, such as the electrical isolation of the synapse, histamine concentration, or Cl^¯^ gradients. The effects of epithelial glia are facilitated by the specific anatomic arrangement of the lamina where epithelial glia share the same synapses with the LMCs and contribute to the greatest extent to the electrical isolation of the photoreceptor-to-LMC synapse.

The functional aspects of the HCLB receptor-mediated modulation of synaptic transmission are not well characterized. The aim of this study was to provide some insight into the functional consequences of this modulation using the following steps: 1) The hypothesis was drawn that HCLB receptors may participate in the process of light adaptation through their effect on the gain of the photoreceptor-to-LMC transmission. To test this, we studied the effects of *hclB* mutations on the intensity-response (V/logI) functions of the ERG responses under different conditions of light adaptation, in the dark as well as under three levels of background illumination. The ERG responses of two *hclB* mutants, *hclB^T1^* (carrying the substitution P293S) and *hclB^T2^* (a null mutant), were compared to those of wild-type flies. 2) Our second task was to establish whether epithelial glia, through its HCLB receptors, participates in the control of the dynamics of sensitivity recovery after short-term light adaptation. For this purpose, we studied the effects of *hclB* mutations on this dynamics.

## Methods

### *Drosophila* stocks

Two *hclB* mutant lines with the genotypes *st hclB^T1^*/TM3, Sb and *st hclB*^T2^/TM3, Sb were generated by mutagenesis with ethyl methanesulfonate. They originated from the Zuker’s collection (Department of Neurosciences, University of California, San Diego, CA). The amino acid substitution (P293S) in *hclB^T1^* mutants affects a highly conserved residue within the second transmembrane domain of the protein, whereas *hclB*^T2^ is a null mutant (W111*) [21]. The flies were kept on yeast-molasses medium at 25 °C with a 12 h:12 h light-dark regime. Because of lack of viable homozygotes from the *hclB^T1^* mutants for all experiments, we used 3–7-day-old hemizygous females produced by crossing of two mutant lines with flies Df(3R)E79/MRS, where the deficiency (86F1–87B9) eliminated the *hclB* chromosomal region. Hemizygous control flies were obtained in a similar way to the wild-type flies Oregon R (Bloomington Drosophila stock center, Bloomington, IN). Here, we follow the nomenclature proposed by [8] for two genes: *hclA* (*ort*) and *hclB*. These are also known as Dm HA-Cl I and Dm HA-Cl II [11], *HisCl* 2 and *HisCl* 1 [10], and *HisCl*-α1 and *HisCl*-α2 [9].

### Electroretinogram recording

ERG was chosen for assessment of sensitivity at the level of photoreceptor-to-LMC synapses because as mentioned above, it represents the activity of both photoreceptors and LMCs [18-20]. Furthermore, as a mass response, it has the advantage of representing the activity of many populations of LMCs with different sensitivities. Because of the high gain of the photoreceptor-to-LMC synapses [23,24], which varies significantly among individual synapses [24], many LMCs with high contrast gain and narrow dynamic range of their responses cover as a population the overall dynamic range of visual responses in particular conditions of ambient illumination.

Flies were briefly anesthetized with CO_2_ and immobilized into Eppendorf pipettes with cut ends, allowing the fly heads to protrude. The heads were additionally fixed to the pipette tips using a small droplet of low-melting wax. The ERGs were recorded by means of glass microelectrodes with a tip diameter of 15–20 μm, filled with Ringer solution (in mmol/l: NaCl 130, KCl 4.7, CaCl_2_ 1.9; MgCl_2_ 4, HEPES 1.3; pH 7.14). The recording electrode was positioned at the corneal surface and the reference electrode was placed onto the head carapace. Conductive electrode gel was used for contact improvement. The ERG responses were amplified at a bandpass of 0–1,000 Hz using a low noise WPI ISO-DAM preamplifier (World Precision Instruments, Sarasota, FL). They were digitized at 5 kHz and analyzed using the WPI LAB-Trax4 Data acquisition system (Data-Trax software, World Precision Instruments).

### Light stimulation

Diffuse light from two green LUXEON^®^V light-emitting diodes (LEDs; LXHL-PMo2; Lumileds Future Electronics, Pointe Claire, Quebec, Canada) with a dominant wavelength of 530 nm were used for test and background illumination. The parameters and the mode of presentation of the test stimuli were controlled by a custom-made LED controller (Stimuled 01, Department of Electronic Engineering, Technical University of Sofia, Bulgaria). The test stimulus intensity was varied over a range of 5.5 log units, the maximal intensity being 7.23 log quanta s^−1^ μm^−2^ at the plane of the eye. The intensities of the backgrounds used were 4.66, 5.66, and 6.66 log quanta s^−1^ μm^−2^. When the intensity-response (V/logI) functions of the ERG responses were tested, each of the eyes was first dark adapted for 2 min and a V/log I function was obtained, with stimulus intensity being increased in 0.5 log unit steps. Then, after 2 min adaptation to each of the backgrounds, V/log I functions were obtained again under the backgrounds. To prove that the functional state of the flies was not changed by the end of the experiments, the eyes were dark adapted again and control V/log I curves were obtained; these did not differ significantly from the first dark adapted curves. To obtain V/logI functions, two types of intermittent stimuli were used: 1) short stimuli with 0.3 s ON and 1.2 s OFF periods; the 0.3 s stimuli were the shortest that allowed for reliable separation and assessment of ON and OFF ERG transients; and 2) long stimuli with 2 s ON and 8 s OFF periods. To test the dynamics of dark sensitivity recovery after short-term light adaptation, 0.3 s ON/1.2 s OFF intermittent stimuli with intensity of 3.73 or 4.73 log quanta s^−1^ μm^−2^ were continuously presented in the dark. Short adapting backgrounds with 2 to 20 s duration and intensities of 5.66, 6.16, or 6.66 log quanta s^−1^ μm^−2^ were added periodically and the amplitudes of the ERG responses preceding and following the light adapting pulses were compared.

### Data analysis

The intensity-response functions of the ERG responses were fitted by the least square method to Naka-Rushton equation, V/V_max_=I^n^/(I^n^+σ^n^), where V represents ERG response amplitude; V_max_, response maximal amplitude; I, test stimulus intensity; σ, stimulus intensity required to produce 0.5 V_max_ response (I_50_); and n, an exponent related to the steepness of the intensity-response function and hence to response dynamic range. The response dynamic range was estimated as the intensity span of the responses with 5 to 95% V_max_ amplitude. The absolute sensitivity of the ERG responses was assessed by their threshold, specifically the stimulus intensity necessary for obtaining a criterion amplitude of 0.5 mV. The response relative sensitivity was assessed by the σ (I_50_) value. For statistical evaluation of the data, two-way ANOVA (ANOVA) with the Bonferroni test (alpha=0.05) was used.

## Results

**Figure 1:**
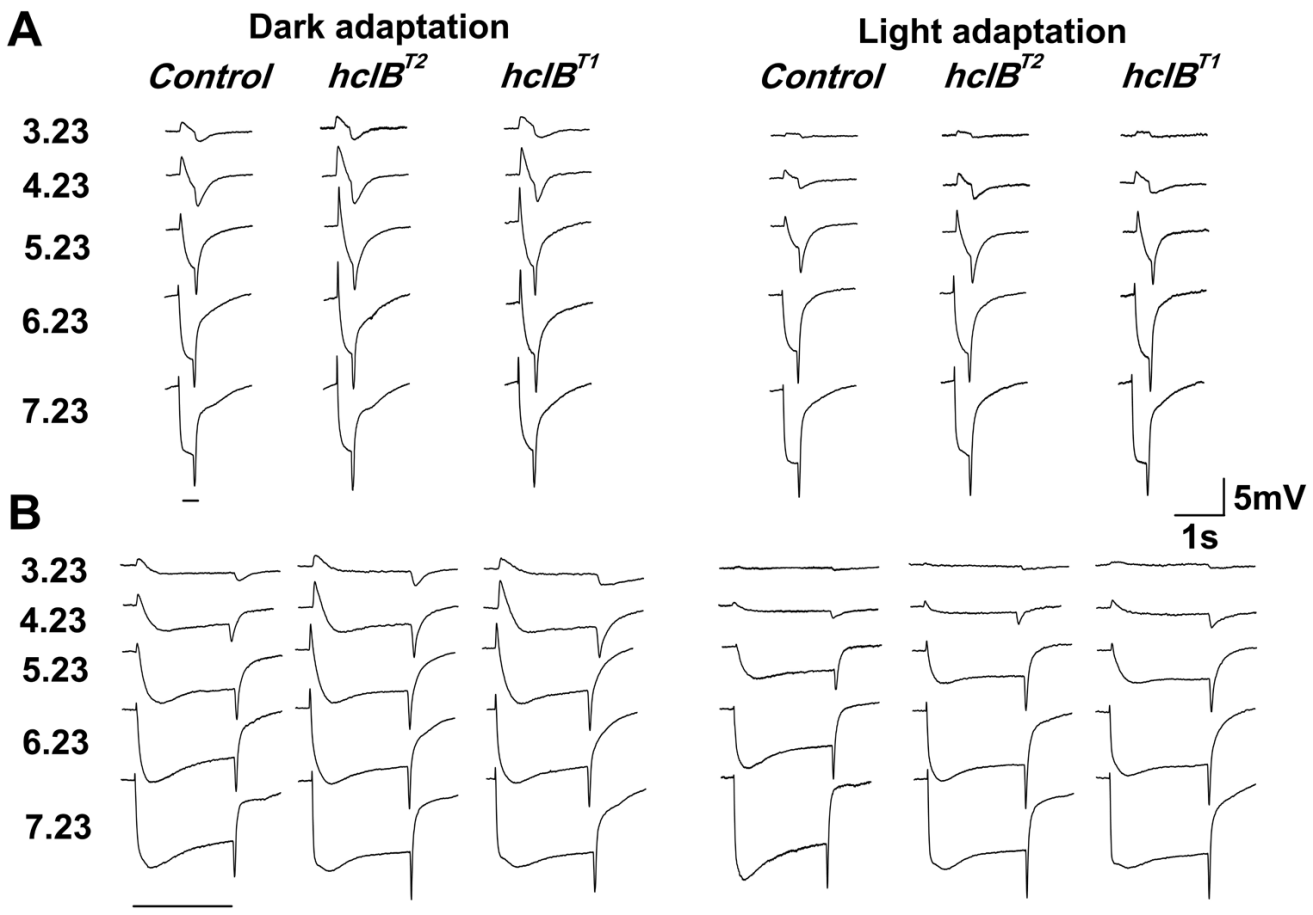
Individual electroretinogram records obtained under different light stimulation conditions from a wild-type fly -*OR/Df(3R)E79*, denoted as control and two *hclB* mutants—*hclBT2/Df(3R)E79*, a null mutant, and *hclBT1/Df(3R)E79*, denoted as *hclB^T2^* and *hclB^T1^*, respectively. In **A** and **B**, electroretinogram (ERG) responses to 0.3 s and 2 s stimuli are represented, respectively. The numbers on the left denote test stimulus intensities (in log quanta s^−1^ μm^−2^). Responses obtained under dark adaptation (left) and light adaptation with a background of 4.66 log quanta s^−1^ μm^−2^ intensity (right) are represented. It is seen that the receptor component of the ERG has similar amplitude in both wild-type and mutant flies, while the ON and OFF transients’ amplitudes are significantly greater in the *hclB* mutants. It can also be observed that the overall duration of the transients is increased in the mutant flies.

The ERGs of the two *hclB* mutants (*hclB^T2^* and *hclB^T1^*) were similar to those of the wild-type flies in that they also consisted of a graded negative (receptor) component and two transient ones—a positive ON and a negative OFF transient, representing, as mentioned above, the activity of the LMCs in the lamina (Figure 1). However, while the amplitude of the receptor component was indistinguishable between the mutant flies and the wild-type controls, the amplitudes of the ERG transients were greater in the two mutants as compared to the wild-type flies (two-way ANOVA with the Bonferroni test; 10^−15^<p<0.05 for ON and OFF responses under different backgrounds, n=10 for all groups of flies in each of the light stimulation conditions). The ERG changes were similar in the two *hclB* mutants. To avoid redundancy, the results for the null mutant *hclB^T2^* are mostly illustrated in the text.

### Intensity-response functions of the electroretinogram responses

#### 1) Responses to 0.3 s stimuli

**Figure 2:**
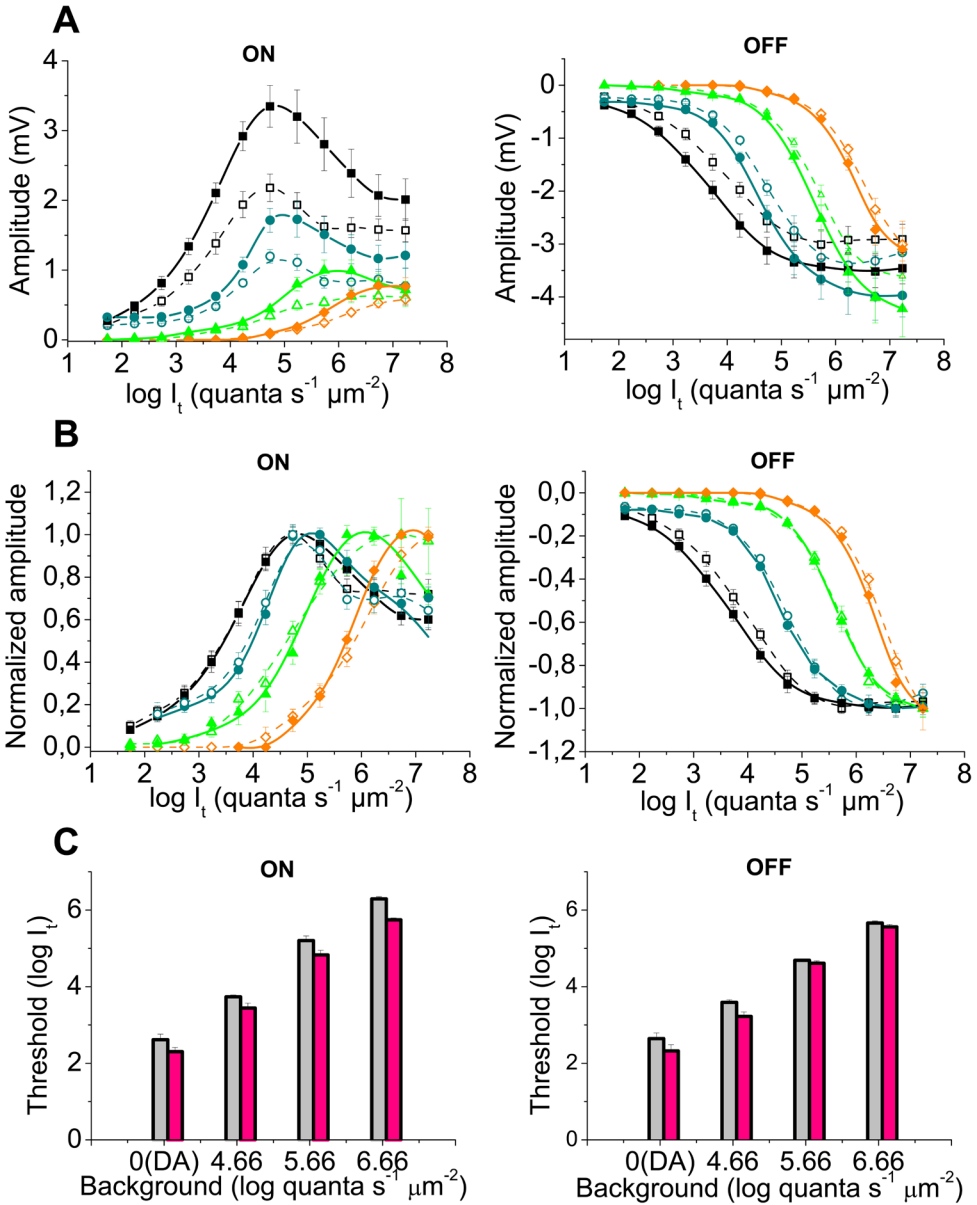
Intensity-response V/logI) functions and thresholds of the electroretinogram responses to 0.3 s stimuli. In **A** and **B**, the V/logI curves of the ON transients (left) and OFF transients (right) are presented obtained in the wild-type flies (open symbols, dashed lines, n=10) and in the null mutant *hclB^T2^* (filled symbols, solid lines, n=10) under dark adaptation (DA, black squares) as well as under three levels of background illumination (4.66 log quanta s^−1^ μm^−2^, blue circles; 5.66 log quanta s^−1^ μm^−2^, green triangles; 6.66 log quanta s^−1^ μm^−2^, orange diamonds). In **A**, the response amplitude in mV versus log stimulus intensity I_t_) is represented. The amplitude of both ON and OFF transients is increased in the *hclB* mutant, the effect of the mutation being more pronounced with respect to ON responses (two way analysis of variance [ANOVA], 10^−15^<p<0.05 for ON and OFF responses under different backgrounds). In **B,** the same functions are normalized to Vmax. The relative sensitivity of the ON and OFF transients, assessed by the I_50_ points of the V/log I curves, is mostly not significantly different between the wild-type and *hclB* mutant flies. The steepness of the V/log I curves of the ON transients is higher in the *hclB* mutant and thus the ON-response dynamic range is narrowed (two way ANOVA, p=0.015). In **C**, the thresholds of the electroretinogram (ERG) ON (left) and OFF (right) transients are presented, obtained under dark adaptation (DA), as well as under three levels of background illumination. The thresholds are estimated using 0.5 mV criterion amplitude. The thresholds of the wild-type flies, (gray columns) and the null mutant *hclB^T2^* (pink columns) are compared. The thresholds of the *hclB^T2^* mutant transients are significantly lower (two way ANOVA, p=1.22×10^−5^ for ON responses; p=0.032 for OFF responses), indicating an increased absolute sensitivity of the mutant responses.

When short (0.3 s ON/ 1.2 s OFF) light stimuli were used, the amplitudes of the ON and OFF transients of the *hclB* mutants were increased under both dark and light adaptation (Figure 1A, Figure 2A). The ON transient increase was greater and approximated 150%–170% of the amplitude obtained in wild-type flies. The OFF transient increase did not exceed 120%. No significant interaction was found with stimulus intensity. Therefore, the absolute sensitivity of the mutant transients was increased, as indicated by their lower thresholds (two-way ANOVA, p=1.22×10^−5^ for ON responses and p=0.032 for OFF responses, n=10 for all groups of flies; Figure 2C), while the relative sensitivity of the transients (assessed by the σ [I_50_] value) was not significantly changed (Figure 2 B). With increasing background intensity and V/logI function steepness, the OFF response threshold diminution became less pronounced (insignificant under the brighter backgrounds). The V/log I curves of the mutant ON transients were slightly steeper than the corresponding curves of the wild-type flies (n value in the Naka-Rushton equation was greater, two way ANOVA, p=0.015) and the dynamic range of the ON transients was thus narrowed by about 0.5 log units.

#### 2) Responses to 2 s stimuli

**Figure 3:**
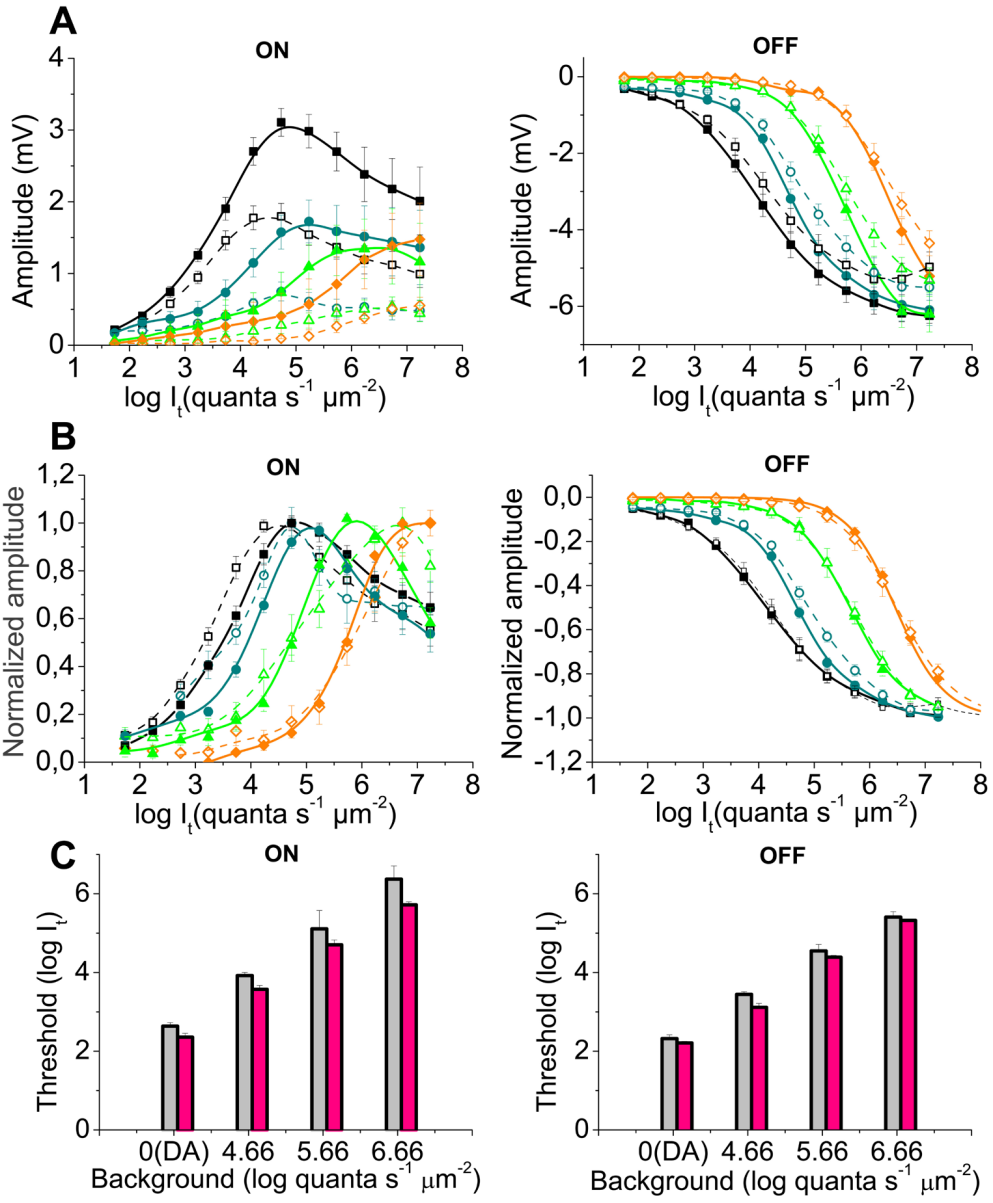
Intensity-response V/logI) functions and thresholds of the electroretinogram responses to 2 s stimuli. In **A** and **B**, the V/logI curves of the ON transients (left) and OFF transients (right) are presented, obtained in the wild-type flies (open symbols, dashed lines, n=10) and in the null mutant *hclB^T2^* (filled symbols, solid lines, n=10) under dark adaptation (DA, black squares) as well as under three levels of background illumination (4.66 log quanta s^−1^ μm^−2^, blue circles; 5.66 log quanta s^−1^ μm^−2^, green triangles; 6.66 log quanta s^−1^ μm^−2^, orange diamonds). In **A**, the response amplitude in mV versus log stimulus intensity I_t_) is represented. The amplitudes of both ON and OFF transients are increased in the *hclB^T2^* mutant (two way analysis of variance [ANOVA], 10^−14^<p<0.01 for ON and OFF responses under different backgrounds), the effect of the mutation being more pronounced with respect to the ON responses. In **B**, the same functions are normalized to Vmax. The relative sensitivity of the ON transients, obtained in *hclB^T2^* flies under dark adaptation as well as under the dimmest background, is decreased (the curves are shifted to the right, p<0.01), while no change in relative sensitivity is observed under the two brighter backgrounds. Thus, in the *hclB* mutant, the shift of the ON transient V/logI curve along the intensity axis during light adaptation occurs within a narrower stimulus intensity range. The ON transient V/logI curves in the light adapted mutant are steeper and the dynamic range is narrowed by about 1 log unit (two way ANOVA, p=0.0035). The relative sensitivity and the dynamic range of the OFF transients are not significantly changed. In **C**, the thresholds of the electroretinogram (ERG) ON (left) and OFF (right) transients are presented obtained under dark adaptation (DA), as well as under three levels of background illumination. The thresholds are estimated using 0.5 mV criterion amplitude. The thresholds of the wild-type flies, (gray columns) and the null mutant *hclB^T2^* (pink columns) are compared. The thresholds of the *hclB^T2^* mutant transients are significantly lower (two-way ANOVA, p=2.34×10^−4^ for ON responses; p=0.026 for OFF responses) indicating an increased absolute sensitivity of the mutant responses.

When long (2 s ON/ 8 s OFF) light stimuli were used, the ON transient increase in the *hclB* mutants was stimulus intensity–dependent under dark adaptation, as well as under the dimmest background used (p=1.57×10^−4^ and p=0.0034 for the two groups, respectively, n=10 for all groups of flies). In these conditions, the amplitude difference between the ON transients of the mutant and wild-type flies was more pronounced in responses to bright stimuli. The amplitudes of these responses in the *hclB* mutants approximated 200%–250% of the corresponding amplitudes in the wild-type controls (Figure 1B, Figure 3A). However, no interaction was found between the ON transient amplitude increase and the test stimulus intensity under the two brighter backgrounds. As a result, the absolute sensitivity of the mutant ON transients was increased (their thresholds were decreased, two way ANOVA, p=2.34×10^−4^, Figure 3C) in all conditions of ambient illumination, while the relative sensitivity of the ON transients was decreased (the V/logI curves were shifted to the right, p<0.01) under dark adaptation and under the lowest background, being unchanged under the two brighter backgrounds (Figure 3B). Thus, the adaptational shift of the ON transient V/logI curve occurred within a narrower intensity range in the mutant flies. The ON transient V/logI curves in the light-adapted mutants were steeper and the dynamic range of these responses was narrowed by about 1 log unit (two way ANOVA, p=0.0035; Figure 3B). Similar to the responses to 0.3 s stimuli, the amplitudes of the OFF transients of the *hclB* mutants approximated 120% of the amplitudes of the corresponding responses in the wild-type flies (two way ANOVA, p=7.11×10^−7^, Figure 3A). The absolute sensitivity of the mutant OFF responses was increased (two way ANOVA, p=0.026 for the decrease of their thresholds; Figure 3C), while the relative sensitivity of these responses was not significantly changed (Figure 3B). The small narrowing of the response dynamic range of the OFF transients was also not significant.

### Temporal characteristics of the electroretinogram responses

**Figure 4:**
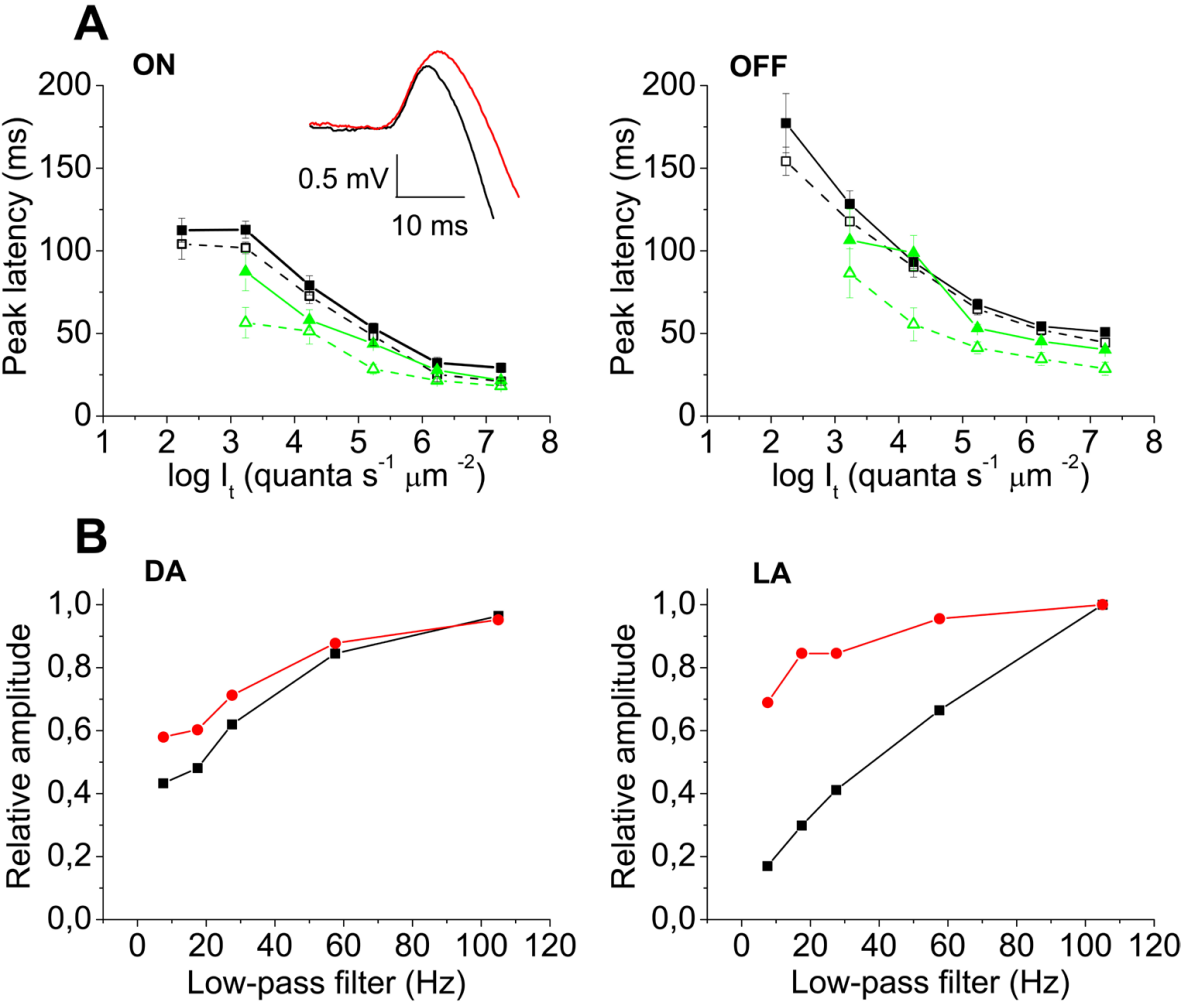
Temporal characteristics of the electroretinogram responses. In **A**, the peak latencies of the electroretinogram (ERG) ON (left) and OFF (right) transients are presented, obtained with 2 s stimuli under dark adaptation (black squares) and under background illumination of 5.66 log quanta s^−1^ μm^−2^ (green triangles). The results obtained in wild-type flies (empty symbols, dashed lines) and in *hclB^T2^* mutants (filled symbols, solid lines) are represented. In the inset, original curves of a wild-type (black) and *hclB^T2^* mutant (red) ON response are superimposed. The beginning of the records corresponds to the stimulus onset. Stimulus intensity=6.73 log quanta s^−1^ μm^−2^. Peak latency is delayed in the *hclB^T2^* mutant (two way analysis of variance [ANOVA], 10^−9^<p<0.05 for different stimulation conditions). The delay is small in the dark-adapted responses, being well pronounced under light adaptation. In **B**, the results of low-pass filtering of the ERG ON transients are presented, obtained using 2 s stimuli in a wild-type fly (black squares) and *hclB^T2^* (red circles) mutant. The amplitudes are normalized to the amplitudes of the nonfiltered signals (raw signals recorded at a bandpass of 0–1000 Hz). Stimulus intensity=6.73 log quanta s^−1^ μm^−2^. On the left, the results obtained under dark adaptation (DA) are presented. On the right, the results obtained under a background of 6.66 log quanta s^−1^ μm^−2^ (light adaptation, LA) are presented. The amplitudes of the mutant responses are decreased to a lesser extent by low-pass filtering. The difference is greater under light adaptation. This is indicative of the slower kinetics of the *hclB* mutant responses and implies that HCLB receptors may contribute to the high-pass filtering of the visual signal during light adaptation.

The temporal characteristics of the ERG transients were also changed in the two *hclB* mutants. The ON and OFF transients had slower time course (see Figure 1 and Figure 4A –inset). The peak latencies of the ON and OFF transients were longer in the mutant as compared to the wild-type flies (10^−9^<p<0.05 for different stimulation conditions, n=10 for all groups of flies in each of the light stimulation conditions; Figure 4A). The difference was small under dark adaptation. Under light adaptation, a well expressed difference was obtained in responses to 2 s stimuli. The changes in the temporal characteristics of the mutant ERG transients were also tested by offline filtering of the ERG records. When low-pass filtered, the responses of the mutant flies were not dramatically reduced, while those of the wild-type flies were strongly diminished (Figure 4B). Conversely, the mutant fly responses were more sensitive to high-pass filtering (result not shown). The difference between the mutant and wild-type flies was more pronounced under light adaptation (Figure 4B, right).

### Time course of dark sensitivity recovery after short-term light adaptation

**Figure 5:**
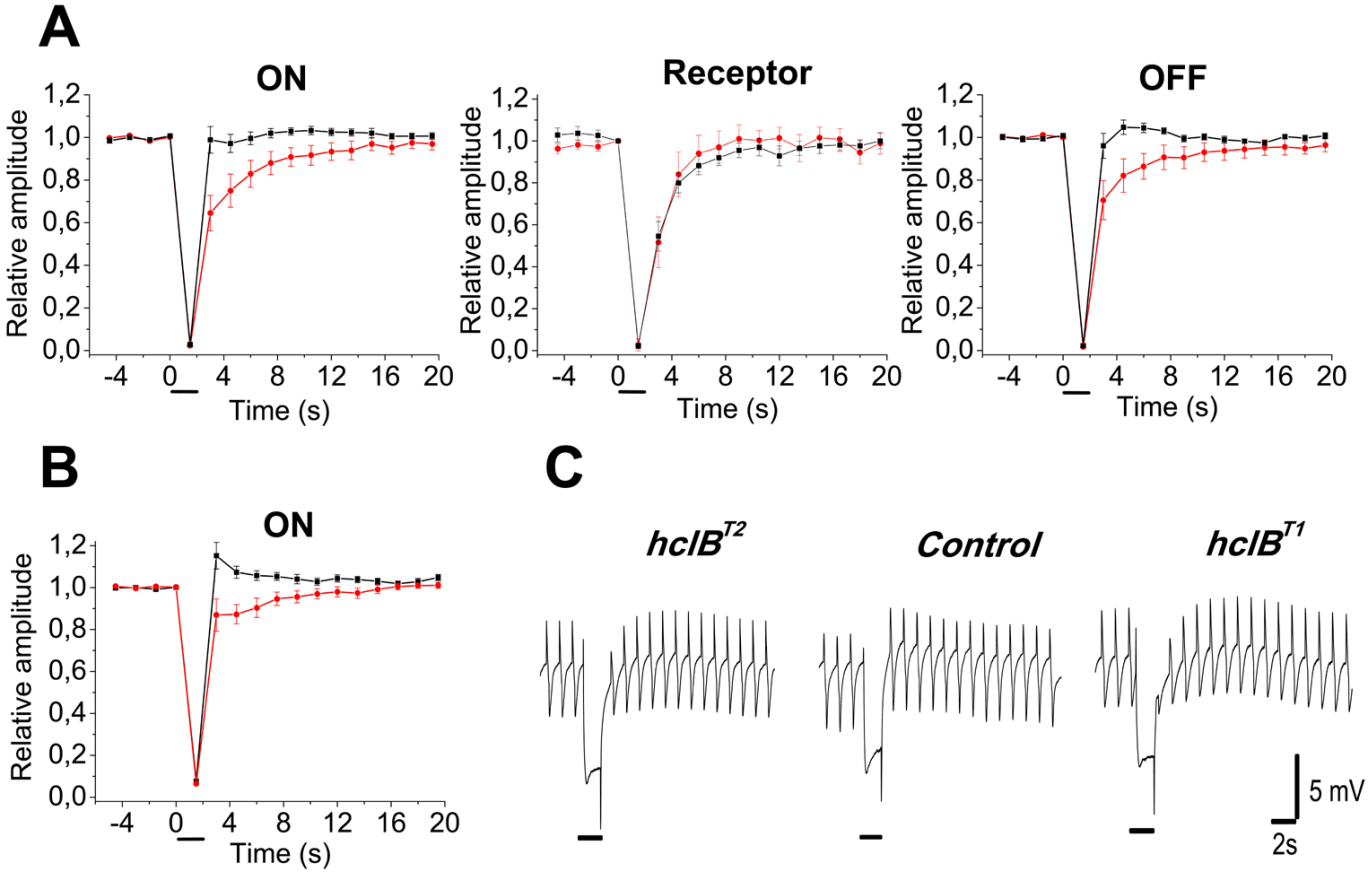
Dynamics of dark sensitivity recovery after short-term light adaptation. A continuous series of 0.3 s stimuli was presented in the dark. Short-time light adaptation was achieved by using 2 s adapting stimuli (denoted by bars under the time scale). **A**: Dynamics of sensitivity recovery of the ON transient (left), ERG receptor component (in the middle) and OFF transient (right) in the wild-type flies (black squares; n=17), and *hclB^T2^* null mutant (red circles; n=15). Test stimulus intensity I_t_)=4.73 log quanta s^−1^ μm^−2^; background intensity I_b_)=6.66 log quanta s^−1^ μm^−2^_._ While instant sensitivity recovery of the ON and OFF transients is seen in wild-type flies, the sensitivity recovery in the *hclB^T2^* mutants is delayed (two way analysis of variance [ANOVA], p=7.42.10^−10^ for the ON response and p=6.8.10^−8^ for the OFF response in these particular conditions). **B**: Postadaptational potentiation of the ON transient in the wild-type-flies (n=22) obtained using the following combination of test and adapting stimuli: I_t_=4.73 log quanta s^−1^ μm^−2^, I_b_=6.16 log quanta s^−1^ μm^−2^. Postadaptational potentiation is lacking in the *hclB^T2^* (n=19) mutant flies. All labels are as in **A**. **C**: Original electroretinogram (ERG) records, obtained from a wild-type control fly (in the middle), *hclB^T2^* mutant (on the left), and *hclB^T1^* mutant (on the right). Stimulation conditions are as in **B**, In the first few seconds after the termination of the 2 s light-adapting stimulus, a postadaptational potentiation is seen in the wild-type fly, while a delayed sensitivity recovery is seen in the two mutants.

The time course of sensitivity recovery of the ERG responses after termination of light adapting stimuli with a few seconds’ duration was assessed by comparing the amplitudes of the ERG responses to 0.3 s stimuli in the periods, preceding and following presentation of adapting stimuli with 2 to 20 s duration and three intensities, specifically Ib1=5.66 log quanta s^−1^ μm^−2^, Ib2=6.16 log quanta s^−1^ μm^−2^, and Ib3=6.66 log quanta s^−1^ μm^−2^. The 0.3 s test stimulus duration was the shortest that allowed for reliable separation between the ON and OFF transients. The following two test stimulus intensities were used: 3.73 and 4.73 log quanta s^−1^ μm^−2^. The first was below the threshold of the ERG receptor component, so that the two ERG transients were recorded in isolation. The second elicited both the receptor component and transients, thus allowing for comparison of sensitivity changes of the receptor and LMC responses. The difference between the *hclB* mutants and the wild-type flies was best manifested when 2 s adapting stimuli were used. Depending on the particular combinations of test and adapting stimuli (the number [n] of the flies in the groups varied between 10 and 20), the following results were obtained: 1) In most cases, in the postadaptational period, the ON and OFF transients of the wild-type flies showed instant sensitivity recovery (Figure 5A). 2) The recovery took several seconds when the dimmer stimulus was combined with a very bright background (not shown). 3) When the brighter test stimulus was combined with Ib1 and Ib2, postadaptational potentiation was observed (Figure 5B,C). The ON transient amplitude increased up to 120% of the preadaptational value, on average, while only a few percent increase of the OFF transient amplitude was observed. The amplitude of the ERG receptor component was significantly diminished during the first few seconds of the postadaptational period in all conditions tested, so in wild-type flies, a clear discrepancy was observed between the dynamics of sensitivity changes of the receptor and postreceptoral ERG components. In the *hclB* mutants, the ON and OFF transient sensitivity recovery was significantly delayed in all conditions tested (two-way ANOVA, with the Bonferroni test, 10^−15^<p<10^−4^ for different combinations of test and adapting stimuli; Figure 5). Some small postadaptational potentiation was only occasionally seen. The dynamics of sensitivity recovery was similar between the receptor and postreceptoral ERG components.

## Discussion

The results of our study show that the glial histamine receptor HCLB participates significantly in visual sensitivity control at the level of the first synapse of the *Drosophila* visual system, as revealed by the effects of mutations in *hclB* on the ERG responses. Our results are the first to demonstrate the effects of *hclB* mutation on the ERG responses in a wide range of light adaptation conditions, specifically in the dark and under three levels of background illumination. The two *hclB* mutants tested—*hclB^T2^* (a null mutant) and *hclB^T1^*—showed similar changes in sensitivity of the ERG responses. In all conditions of ambient illumination, the ERG receptor component was not significantly different between the mutant and wild-type flies. At the same time, the amplitude of both ON and OFF transients was greater in the *hclB* mutants and the absolute sensitivity of these responses, assessed by their 0.5 mV threshold, was increased. The ON responses were affected to a greater extent. These results are consistent with our previous findings [21,22], and for ON transients, with the results of other authors [12] obtained under dark adaptation only. They indicate that the effects of the photoreceptor neurotransmitter, histamine, mediated by the HCLB receptors of epithelial glia in the wild-type flies, change (decrease) the gain of the synaptic transmission from photoreceptors to LMCs in the lamina, thus decreasing visual sensitivity. The question then arises of whether this sensitivity decrease contributes to the process of light adaptation.

### Contribution of the influences mediated through the histamine receptor encoded by the gene *hclB* to the light adaptation process

The light adaptation process allows the visual system to work under a wide range of ambient illumination (~10 log units), regardless of the restricted range of visual cell responses. It changes visual sensitivity to prevent response saturation while keeping the contrast sensitivity constant (independent of the ambient illumination) [25,26]. This is achieved by a shift of the visual operating range along the stimulus intensity axis (demonstrated by the almost parallel shift of the response V/logI curves). This mechanism already operates at the photoreceptor level, but postreceptoral mechanisms are also involved. The process of light adaptation is also characterized by changes in the temporal characteristics of the transmitted signals such that the redundant (low-frequency) information is filtered, which increases temporal resolution and optimizes encoding the dynamic properties of visual stimuli [24,27,28]. This raises the following question: Are the histamine-gated HCLB receptors involved in the above mentioned processes? Our results show that 1) under light adaptation, the ON transients of the *hclB* mutants saturate earlier. Their dynamic range is narrowed (in our experiments by 0.5 log units for 0.3 s stimuli and by 1 log unit for 2 s stimuli). This means that, under light adaptation, the functional HCLB receptors contribute to avoid saturation and widen the dynamic range of the laminar ON responses. The same is not true, however, for the OFF responses, whose dynamic range is not significantly changed in the *hclB* mutants. 2) In the *hclB* mutants, the relative sensitivity of the ON responses to long stimuli is decreased (the V/logI curves are shifted to the right) under dark adaptation as well as under moderate-intensity backgrounds, while the relative sensitivity of the responses obtained under bright backgrounds is not changed. Therefore, the intensity span of the V/logI curve shift along the intensity axis during light adaptation is narrowed in the mutant flies. This implies that the functional HCLB receptors have some contribution to this shift. This effect is particularly true when long light stimuli are presented. Again, a similar effect is not observed with respect to the OFF response. 3) The *hclB* mutant flies have slower kinetics of their ON and OFF transients, indicated by the delayed peak latencies of these responses. This result is consistent with the data, obtained at a cellular level, showing slower kinetics of the mutant LMCs’ responses to light flashes [12]. The slower kinetics of the mutant ON and OFF transients is also indicated by the fact that they are less sensitive to low-pass filtering and more sensitive to high-pass filtering. The effect of *hclB* mutation on the peak latency is most prominent in responses to long stimuli under light adaptation. The effect of filtering is also more pronounced under light than dark adaptation. This implies that the HCLB receptors contribute to the filtering of low-frequency components of visual signals during light adaptation (redundancy reduction). On the basis of the results presented above, a conclusion may be drawn that the histamine-gated HCLB receptors have some contribution to the process of light adaptation, which is more pronounced with respect to the ON response. Concerning OFF response, the effects of light adaptation on temporal characteristics are mainly affected.

### Diverse mechanisms may underlie the sensitivity changes mediated by the histamine receptor encoded by the gene *hclB*

What are the possible mechanisms involved in the HCLB-mediated modulation of visual responses mentioned above? The HCLB receptor is expressed in the epithelial glia [12,13], which separates the individual cartridges in the lamina, participating in the creation of a high-resistance barrier [14-16]. Along with LMCs, the epithelial glia is a postsynaptic element in the tetrad synapses, which are the most numerous synaptic contacts of R1-R6 type photoreceptors in the first optic neuropile [14,16,17]. Epithelial glia is an astrocyte-type glia, so it is involved in regulation of electrolyte homeostasis of the extracellular space, as well as in neurotransmitter clearance and recycling [29-32]. As the tetrad synapses are invaginated synapses, they are characterized by a narrow synaptic cleft and high extracellular electrical resistance. This facilitates changes in neurotransmitter and electrolyte concentrations and the creation of electrical field potentials. Several mechanisms might thus be involved in the HCLB receptor–mediated modulation of synaptic transmission at the photoreceptor-to-LMC synapse. Some of these have been discussed elsewhere [12,32]. 1) HCLB receptors may effectively compete with HCLA receptors for histamine, released by photoreceptors upon light stimulation. They have been shown to be even more sensitive to histamine than the homomeric HCLA receptors, expressed in LMCs [10,12]. 2) Histamine binding to HCLB may activate histamine uptake by epithelial glia and thus change the histamine concentration in the synaptic cleft. 3) As HCLB receptors are Cl¯ channels, the ion currents through these channels may shunt the postsynaptic Cl¯ currents in LMCs. This is possible if the Cl¯ equilibrium potential is more negative than the glial membrane potential and the histamine-gated HCLB channel opening results in inward Cl¯ currents. The membrane potential of glial cells is highly dependent on K^+^ permeability and is thus strongly negative. Indeed, an intracellular potential of about –90 mV has been recorded in laminar glia with respect to the intercellular space of the retina [33]. However, if we take into account the strongly negative value of the extracellular field potential in the laminar cartridges (−20 to −40 mV [28,33]), the transmembrane potential of the epithelial glia in the laminar cartridges may well be within the range of −50 to −70 mV and positive in reference to E_Cl¯._ This assumption is supported by the fact that laminar glial cells produce slow hyperpolarization of a few mV during light illumination [33]. 4) Inward Cl^¯^ currents through HCLB channels may be a source of the depolarizing shift of the extracellular field potential in the cartridges during illumination, similar to the postsynaptic currents in the LMCs, a mechanism proposed in [28]. It has been suggested by several authors [15,16,24,28,34,35] that changes in the laminar extracellular field potential may be strongly involved in the process of light adaptation because of the resultant changes in membrane potential of photoreceptor axon terminals and thus the neurotransmitter release. They also may take part in the modulation of temporal characteristics of the signal transmitted through the photoreceptor-to-LMC synapse by subtracting low-frequency components of the signal. This mechanism is similar to that proposed for the horizontal cell-to-photoreceptor feedback in vertebrate retina [36,37]. 5) Cl^¯^ currents through HCLB channels can change the extracellular Cl^¯^ concentration, and thus the Cl^¯^ electrochemical gradient and postsynaptic currents in the LMCs and amacrine cells. The effect of inward Cl^¯^ currents in LMCs and glial cells is opposed by the effect of Cl^¯^ extrusion mechanisms in these cells. As shown in [38], these ion fluxes may not be balanced, which may modulate visual responses of LMCs. This mechanism will be discussed further below.

### Effects on the dynamics of sensitivity changes after short-term light adaptation, mediated through the histamine receptor encoded by the gene *hclB*

Our results are the first to show the involvement of the histamine receptor HCLB in the control of dynamics of dark sensitivity recovery after short-term light adaptation. They clearly demonstrate that HCLB receptors speed up the sensitivity recovery after cessation of light-adapting stimuli with a duration of a few seconds. They may also participate in potentiation of the LMC responses in the postadaptational period. In our experiments, the dynamics of sensitivity changes was tested in light adaptation conditions similar to those reported previously in the blowfly [38]. The authors of this research followed the changes in the resting potential and responses of the photoreceptors and LMCs to light flashes after cessation of an adapting illumination with 100 ms to 20 s duration. They found a small depolarization and potentiation of the LMCs’ light responses in the postadaptational period lasting up to 20 s (postadaptational potentiation). During the postadaptational period, photoreceptors were shown to undergo hyperpolarization and an increase of their light response amplitude. However, this process had a slower time course (Figure 5 in their paper) and in the first few seconds of the postadaptational period, the potentiation of the LMCs responses was not in concert with the changes in photoreceptor responses. A mechanism was proposed and supported by the results of ionophoretic injections of Cl^¯^ whereby the postadaptational potentiation is at least partially due to the imbalance between the reduced inward Cl^¯^ current through histamine-gated Cl^¯^ channels in LMCs after cessation of light stimulation and the increased outward Cl^¯^ current created by activation of Cl^¯^ extrusion mechanisms (because of Cl^¯^ accumulation during the preceding adaptation). The resulting change in E_Cl¯_ was supposed to underlie, at least partially, the potentiation of the LMCs’ responses. The histamine-gated Cl^¯^ channels that are expressed on LMCs and are thus supposed to participate in the above mentioned mechanism are HCLA receptors [12,13]. It seems plausible, however, that a similar mechanism may take place in the epithelial glia, which are also postsynaptic elements in tetrad synapses, with the participation of the HCLB receptors. Indeed, our results support this hypothesis. In wild-type flies, we found a postadaptational potentiation of the ERG ON and OFF transients, which depended in the same manner on background intensity and duration and which followed a similar time course to that described for LMCs [38]. At the same time, the ERG receptor component was diminished and recovered in a few seconds. The discrepancy between the sensitivity changes in the ERG receptor and laminar components in the wild-type flies observed in our experiments was similar to the discrepancy between the sensitivity changes in photoreceptor and LMC responses during the first few seconds of the postadaptational period (but not later on) seen in [38]. Potentiation was absent or only rarely seen in the *hclB* mutants; the dark sensitivity recovery was delayed and the postadaptational changes in the amplitude of the ERG transients were similar to those of the photoreceptor component.

The delayed sensitivity recovery in the *hclB* mutants might also be a possible explanation of the difference in the degree of potentiation of their ERG transients, depending on the type of stimulation, namely, 0.3 s ON/1.2 s OFF or 2 s ON/8 s OFF, the potentiation being more pronounced in the second case. Although in both cases the ON/OFF period duration ratio was the same, the delayed dynamics of sensitivity changes in the *hclB* mutants resulted in better recovery and greater sensitivity when longer stimuli were presented at longer interstimulus intervals.

### Comparison of the influences mediated through the histamine receptor encoded by the gene *hclB* on the ON and OFF responses

The results of this study, as well as those of [12], show that mutations in *hclB* produce more pronounced effects on the ERG ON as compared to OFF transient. A question arises as to why the OFF responses of the second-order neurons, represented by the ERG OFF transient, are less affected by the HCLB receptor/channel activation. A possible explanation may be that in LMCs, the closure of the histamine-gated Cl^¯^ channels is not the only mechanism for OFF response generation. It has been shown that a conductance increase rather than decrease was observed during LMC OFF response generation [39,40], and that mutations in certain genes can separately affect the ON and OFF responses of LMCs [19].

### Concluding remarks

The existence of two types of receptors—a neuronal (HCLA) and a glial one (HCLB)—for the photoreceptor neurotransmitter histamine is an important mechanism for precise control of synaptic transmission in the photoreceptor synapse in the optic lamina of *Drosophila*. Although not directly involved in forward transmission of the visual signal, the glial receptor HCLB can significantly affect synaptic gain and response dynamics through changes in the synaptic environment, such as the electrical isolation of the synapse, neurotransmitter concentration, or Cl^¯^ gradients. Comparing the ERG responses of *hclB* mutants, which lack functional HCLB receptors, to those of wild-type flies, we were able to characterize some functional consequences of the HCLB receptor-mediated modulation of synaptic transmission. The results of our study demonstrate that the effects of histamine, mediated by the glial receptor HCLB, contribute to the process of light adaptation, which helps avoid saturation and produce dynamic responses to a wide range of stimulus intensities under different levels of ambient illumination. The HCLB-mediated effects also speed up the sensitivity recovery after short-term light adaptation (such as after looking at a bright object in the visual scene) and contribute to the mechanism of postadaptational potentiation. By speeding up the time course of visual responses, HCLB receptors help improve the temporal resolution of the visual system and reduce the redundant (low-frequency) information, which is important for information encoding. As a whole, the results of this study support the concept of the important contribution of glial cells in synaptic transmission and information processing in the central nervous system.

## References

[1] Elias MS, Evans PD (1983). Histamine in the insect nervous system: distribution, synthesis and metabolism.. J Neurochem.

[2] Hardie RC (1987). Is histamine a neurotransmitter in insect photoreceptors?. J Comp Physiol.

[3] Sarthy PV (1991). Histamine: a neurotransmitter candidate for *Drosophila* photoreceptors.. J Neurochem.

[4] Melzig J, Burg M, Gruhn M, Pak W, Buchner E (1998). Selective Histamine Uptake Rescues Photo- and Mechanoreceptor Function of Histidine Decaroxylase-Deficient *Drosophila* Mutant.. J Neurosci.

[5] Stuart AE (1999). From fruit flies to barnacles, histamine is the neurotransmitter of arthropod photoreceptors.. Neuron.

[6] Hardie RC (1989). A histamine-activated chloride channel involved in neurotransmission at a photoreceptor synapse.. Nature.

[7] Skingsley DR, Laughlin SB, Hardie RC (1995). Properties of histamine-activated chloride channels in the large monopolar cells of the dipterian compound eye: a comparative study.. J Comp Physiol A Neuroethol Sens Neural Behav Physiol.

[8] Gengs C, Leung HT, Skingsley DR, Iovchev MI, Yin Z, Semenov EP, Burg MG, Hardie RC, Pak WL (2002). The target of *Drosophila* photoreceptor synaptic transmission is a histamine-gated chloride channel encoded by *ort* (*hclA*).. J Biol Chem.

[9] Gisselmann G, Pusch H, Hovemann BT, Hatt H (2002). Two cDNAs coding for histamine-gated ion channels in *D. melanogaster.*. Nat Neurosci.

[10] Zheng Y, Hirschberg B, Wang AP, Hunt DC, Ludmerer SW, Schmatz DM, Cully DF (2002). Identification of two novel *Drosophila melanogaster* histamine-gated chloride channel subunits expressed in the eye.. J Biol Chem.

[11] Witte I, Kreienkamp HJ, Gewecke M, Roeder T (2002). Putative histamine-gated chloride channel subunits of the insect visual system and thoracic ganglion.. J Neurochem.

[12] Pantazis A, Segaran A, Liu CH, Nikolaev A, Rister J, Thum AS, Roeder T, Semenov E, Juusola M, Hardie RC (2008). Distinct roles for two histamine receptors (*hclA* and *hclB*) at the *Drosophila* photoreceptor synapse.. J Neurosci.

[13] Gao S, Takemura SY, Ting CY, Huang S, Lu Z, Luan H, Rister J, Thum AS, Yang M, Hong ST, Wang JW, Odenwald WF, White BH, Meinertzhagen IA, Lee CH (2008). The neural substrate of spectral preference in *Drosophila.*. Neuron.

[14] Meinertzhagen IA, Sorra KE (2001). Synaptic organisation in the fly’s optic lamina: few cells, many synapses and divergent microcircuits.. Prog Brain Res.

[15] Shaw SR (1975). Retinal resistance barriers and electrical lateral inhibition.. Nature.

[16] Shaw SR (1984). Early visual processing in insects.. J Exp Biol.

[17] Meinertzhagen IA, O'Neil SD (1991). Synaptic organization of columnar elements in the lamina of the wild type in *Drosophila melanogaster.*. J Comp Neurol.

[18] Alawi AA, Pak W (1971). On-Transient of Insect Electroretinogram: Its Cellular Origin.. Science.

[19] Heisenberg M (1971). Separation of receptor and lamina potentials in the electroretinogram of normal and mutant *Drosophila.*. J Exp Biol.

[20] Coombe PE (1986). The large monopolar cells L1 and L2 are responsible for ERG transients in *Drosophila.*. J Comp Physiol A Neuroethol Sens Neural Behav Physiol.

[21] Yusein S, Velikova N, Kupenova P, Hardie R, Wolstenholme A, Semenov E (2008). Altered ivermectin pharmacology and defective visual system in *Drosophila* mutants for histamine receptor HCLB.. Invert Neurosci.

[22] Yusein S, Wolstenholme A, Semenov E (2010). Functional consequences of mutations in the *Drosophila* histamine receptor HCLB.. J Insect Physiol.

[23] Laughlin SB, Howard J, Blakeslee B (1987). Synaptic limitations to contrast coding in the retina of the blowfly *Calliphora.*. Proc R Soc Lond B Biol Sci.

[24] Juusola M, Uusitalo RO, Weckström M (1995). Transfer of graded potentials at the photoreceptor-interneuron synapse.. J Gen Physiol.

[25] Shapley R, Enroth-Cugell C. Visual Adaptation and Retinal Gain Controls. In: N. Osborne, G. Chader editors. Progress in Retinal Research, 1984. Vol. 3, p. 263–346, Pergamon Press (New York)

[26] Laughlin SB (1989). The role of sensory adaptation in the retina.. J Exp Biol.

[27] Zheng L, Nikolaev A, Wardill T, O’Kane CJ, de Polavieja GG, Juusola M (2009). Network Adaptation Improves Temporal Representation of Naturalistic Stimuli in *Drosophila* Eye: I Dynamics.. PLoS ONE.

[28] Weckström M, Laughlin S (2010). Extracellular Potentials Modify the Transfer of Information at Photoreceptor Output Synapses in the Blowfly Compound Eye.. J Neurosci.

[29] Borycz J, Borycz JA, Loubani M, Meinertzhagen IA (2002). *tan* and *ebony* Genes Regulate a Novel Pathway for Transmitter Metabolism at Fly Photoreceptor Terminals.. J Neurosci.

[30] Richardt A, Rybak J, Störtkuhl KF, Meinertzhagen IA, Hovemann BT (2002). Ebony protein in the Drosophila nervous system: optic neuropile expression in glial cells.. J Comp Neurol.

[31] Stuart AE, Borycz J, Meinertzhagen IA (2007). The dynamics of signaling at the histaminergic photoreceptor synapse of arthropods.. Prog Neurobiol.

[32] Edwards TN, Meinertzhagen IA (2010). The functional organisation of glia in the adult brain of *Drosophila* and other insects.. Prog Neurobiol.

[33] Zheng L, de Polavieja GG, Wolfram V, Asyali MH, Hardie RC, Juusola M (2006). Feedback network controls photoreceptor output at the layer of first visual synapses in *Drosophila.*. J Gen Physiol.

[34] Laughlin SB (1974). Neural integration in the first optic neuropile of dragonflies.. J Comp Physiol A Neuroethol Sens Neural Behav Physiol.

[35] Laughlin SB, Hardie R (1978). C. Common strategies for light adaptation in the peripheral visual systems of fly and dragonfly.. J Comp Physiol A Neuroethol Sens Neural Behav Physiol.

[36] Byzov AL, Shura-Bura TM (1986). Electrical feedback mechanism in the processing of signals in the outer plexiform layer of the retina.. Vision Res.

[37] Kamermans M, Fahrenfort I, Schultz K, Janssen-Bienhold U, Sjoerdsma T, Weiler R (2001). Hemichannel-Mediated Inhibition in the Outer Retina.. Science.

[38] Uusitalo RO, Weckström M (2000). Potentiation in the First Visual Synapse of the Fly Compound Eye.. J Neurophysiol.

[39] Guy RG, Srinivasan MV (1988). Integrative properties of second-order visual neurons: a study of large monopolar cells in the dronefly *Eristalis.*. J Comp Physiol A Neuroethol Sens Neural Behav Physiol.

[40] Laughlin SB, Osorio D (1989). Mechanisms for neural signal enhancement in the blowfly compound eye.. J Exp Biol.

